# Leukoaraiosis, intracerebral hemorrhage, and functional outcome after acute stroke thrombolysis

**DOI:** 10.1212/WNL.0000000000003605

**Published:** 2017-02-14

**Authors:** Kannikar Kongbunkiat, Duncan Wilson, Narongrit Kasemsap, Somsak Tiamkao, Fatima Jichi, Vanessa Palumbo, Michael D. Hill, Alastair M. Buchan, Simon Jung, Heinrich P. Mattle, Nils Henninger, David J. Werring

**Affiliations:** From the Stroke Research Centre (K.K., D.W., D.J.W.), Department of Brain Repair and Rehabilitation, UCL Institute of Neurology and The National Hospital for Neurology and Neurosurgery, Queen Square, London, UK; Division of Neurology, Department of Medicine, Faculty of Medicine (K.K., N.K., S.T.), and North-Eastern Stroke Research Group (K.K., N.K., S.T.), Khon Kaen University, Thailand; UCL School of Life & Medical Sciences (F.J.), London, UK; Stroke Unit (V.P.), Department of Neurology, Careggi University Hospital, Florence, Italy; Hotchkiss Brain Institute (M.D.H.), Cumming School of Medicine, University of Calgary, Canada; Radcliffe Department of Medicine (A.M.B.), University of Oxford, John Radcliffe Hospital, UK; Department of Neurology (S.J., H.P.M.), Inselspital, University of Bern, Switzerland; and Departments of Neurology and Psychiatry (N.H.), University of Massachusetts Medical School, Worcester.

## Abstract

**Objective:**

To perform a systematic review and pooled meta-analysis of published studies to assess whether the presence of leukoaraiosis on neuroimaging before treatment with thrombolysis (IV or intra-arterial) is associated with an increased risk of symptomatic intracerebral hemorrhage (sICH) or poor functional outcome.

**Methods:**

We included studies of patients with acute ischemic stroke, treated with IV or intra-arterial thrombolysis, which assessed functional outcome (3-month modified Rankin Scale [mRS]) or sICH in relation to leukoaraiosis on pretreatment neuroimaging (CT or MRI). We used random-effects models to calculate pooled relative risks (RR) of sICH and poor functional outcome (mRS ≥ 2) for any vs no leukoaraiosis (using any rating scale) and for no to mild vs moderate to severe leukoaraiosis (using the Van Swieten or Fazekas Schmidt scale).

**Results:**

We identified 15 studies (total n = 6,967). For sICH outcome, the RR was 1.65 (n = 5,551; 95% confidence interval [CI] 1.26–2.16, *p* = 0.001) with an absolute risk (AR) increase of 2.5% for any leukoaraiosis vs none. The RR was 2.4 (n = 4,192; 95% CI 1.83–3.14, *p* = 0.001) with an AR increase of 6.2% for moderate to severe vs no to mild leukoaraiosis. For poor functional outcome; the RR was 1.30 (n = 3,401; 95% CI 1.19–1.42, *p* = 0.001) with an AR increase of 15.4% for any leukoaraiosis vs none. The RR was 1.31 (n = 3,659; 95% CI 1.22–1.42, *p* = 0.001) with an AR increase of 17.5% for moderate to severe vs no to mild leukoaraiosis. No statistical heterogeneity was noted for any of the analyses.

**Conclusions:**

Leukoaraiosis presence and severity are consistently associated with an increased risk of sICH and poor functional outcome after IV or intra-arterial thrombolysis for acute ischemic stroke.

Thrombolytic therapy in acute ischemic stroke increases the risk of intracerebral hemorrhage (ICH).^1^ Symptomatic ICH (sICH) occurs in 2.4%–10% of patients within 24–36 hours after thrombolysis^2–4^ and is associated with an increased risk of subsequent death or disability.^5^ Imaging markers of small vessel disease, e.g., leukoaraiosis (LA) and cerebral microbleeds, are potential risk factors for thrombolysis-related ICH^6,7^ and poor clinical outcome.^8,9^

On plain CT, LA is denoted by bilateral (patchy or diffuse) areas of hypodensities in the periventricular white matter regions, which may extend to the centrum semiovale.^10^ On MRI, LA is seen on T2-weighted magnetic sequences as hyperintense signal, termed white matter hyperintensities of presumed vascular origin.^10^ LA is primarily attributed to cerebral small vessel disease, causing chronic ischemic damage through disturbance of the microcirculation; LA has variable severity but typically progresses gradually over time, with the accumulation of vascular risk factors and advancing age.^7^ Data regarding the association between LA and sICH have been conflicting, with some studies reporting an increased risk of sICH and poor clinical outcome following thrombolysis for acute ischemic stroke,^7,9,11–14^ and others not.^15–18^ We therefore performed a systematic review and pooled meta-analysis of published studies to assess the evidence whether the presence of LA on neuroimaging before treatment with thrombolysis (IV/intra-arterial) is associated with an increased risk of sICH and poor functional outcome.

## METHODS

### Search strategy and selection criteria.

We searched MEDLINE and EMBASE between January 1, 1995, and September 1, 2015, using the following search terms: leukoaraiosis or white matter or small vessel disease(s) (SVD) in association with thromboly* or tPA or tissue plasminogen activator or fibrinoly*. We identified further studies from reference lists from publications and identified from the above search terms. We did not limit the search to any language. Case reports were excluded.

### Eligibility criteria.

Prospective or retrospective studies, which assessed sICH risk or functional outcome and rated LA on pretreatment CT or MRI in patients with acute ischemic stroke treated with thrombolysis, were included.

### Data extraction.

Two authors (K.K. and D.W.) read the abstracts of selected potentially relevant studies, reviewed the full text of those that appeared relevant, and extracted data independently. Both authors identified and read each study and resolved any differences by discussion with a third author (D.J.W.). We extracted information on type of study, number and characteristics of participants (including mean age and sex), neuroimaging parameters, duration of follow-up, number of participants with LA, number of participants with the outcome event (sICH defined according to standard criteria), and functional outcome. Consensus on the final studies included was reached by discussion. Where data were not available on LA, sICH, and the functional outcome, we contacted the study authors to request aggregate-level data. A flow chart of study selection is provided in figure e-1 at Neurology.org. Where all appropriate data were not extractable from the published articles, we contacted each study group to provide all required data for our analysis.

### Quality assurance.

We followed the Strengthening the Reporting of Observational Studies in Epidemiology as quality indicators^19^ and we prepared the article with reference to Meta-analyses of Observational Studies guidelines.^20^

### LA definition and statistical analysis.

LA severity was defined using various scales: the Gorter scale^21^; the van Swieten scale^22^; the Blennow rating scale^23^; the Wahlund rating scale^24^; the Fazekas scale^25^; the Fazekas and Schmidt (2003)^26^ scale; the Fazekas and Schmidt (1992) scale^27^; and the Scheltens score scale.^28^ Although combining scales potentially introduces heterogeneity, the good correlation for presence/absence and different severity grades among the different visual rating scales allows meaningful comparison of the results.^29^ Based on the findings reported, we first compared outcomes between the presence of any LA vs no LA; second, we compared outcomes between no to mild LA vs moderate to severe LA. We pooled data for both of these comparisons in separate meta-analyses.

For the sICH outcome (no LA vs any LA), we were able to combine all of the different scales, as no LA was defined the same regardless of scale (10 articles).^11–18,30,31^ Eight studies provided sICH risk between patients with no to mild LA vs moderate to severe LA; 2^6,32^ of 8 studies used the Fazekas and Schmidt 2003 (scores 0–1 defined as no to mild and 2–3 as moderate to severe). A total of 6^7,12–14,16,31^ of 8 studies used van Swieten (scores 0–2 defined as no to mild and 3–4 defined as moderate to severe).

For functional outcome at 3 months, the same analyses as above were undertaken: no vs any LA (8 articles)^9,13–16,18,31,33^ and no to mild vs moderate to severe (van Swieten^22^) (6 articles).^7,9,13,14,16,31^

We used a random-effects model to calculate the pooled relative risk (RR) of sICH and functional outcome in patients with vs without LA; weights were calculated using the inverse variance method. We assessed heterogeneity using I^2^ and χ^2^ statistics indicating the percentage of variance in a meta-analysis attributable to study heterogeneity and through visual inspection of the forest plots. We explored publication bias with funnel plots. We calculated pooled absolute risk between each group comparing any LA vs no LA and moderate to severe LA vs no to mild LA. Meta-analyses were performed using Stata 11.2 (StataCorp LP, College Station, TX).

## RESULTS

We identified 667 articles in our initial search of MEDLINE and EMBASE; 19 studies met our inclusion criteria. Nine articles provided the required data and we e-mailed the authors of the remaining 10 articles to obtain the required data. Four articles (831 patients in total) were unable to provide functional outcome data. We included data from 15 articles including a total of 6,967 patients. The number of patients with LA was 2,934, giving an overall pooled prevalence of 42.1%. A flow chart of study selection is shown in figure e-1. A summary of the characteristics of included studies is provided in tables e-1–e-5. Not all LA measures or outcomes were available from all studies; therefore different numbers of participants contributed to each analysis as described in detail below.

### sICH risk.

**Figure 1:**
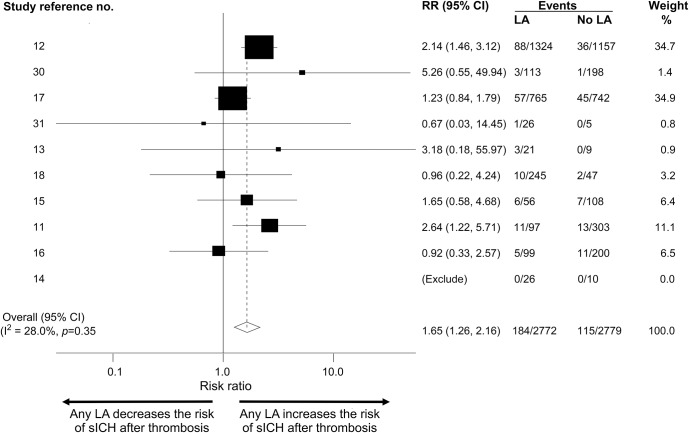
Forest plot of symptomatic intracerebral hemorrhage (sICH): Any leukoaraiosis (LA) vs no LA Forest plot of sICH-associated LA in post-thrombolysis patients: any LA vs no LA. CI = confidence interval; RR = relative risk.

For this analysis, we included data from 10 articles^11–18,30,31^ including 5,551 patients comparing sICH risk post thrombolysis between patients with no LA vs any LA. The total sICH rate was 299/5,551 (5.4%). The sICH event rate in those with any LA was 6.6% vs 4.1% for those without LA; thus, any LA confers an absolute risk increase of 2.5% for sICH. The pooled RR of sICH risk was 1.65 (95% confidence interval [CI] 1.26–2.16; *p* = 0.001) in those with LA vs those without (figure 1). Heterogeneity (I^2^) was 28.0% between the studies. In meta-regression, 3 factors accounted for the heterogeneity as assessed by I^2^: initial stroke severity (NIH Stroke Scale score), type of imaging (CT or MRI), and use of endovascular treatment. However, none of these variables was statistically associated with the outcome of interest (sICH). There was no evidence of publication bias in the funnel plot (figure e-2).

**Table 1:**
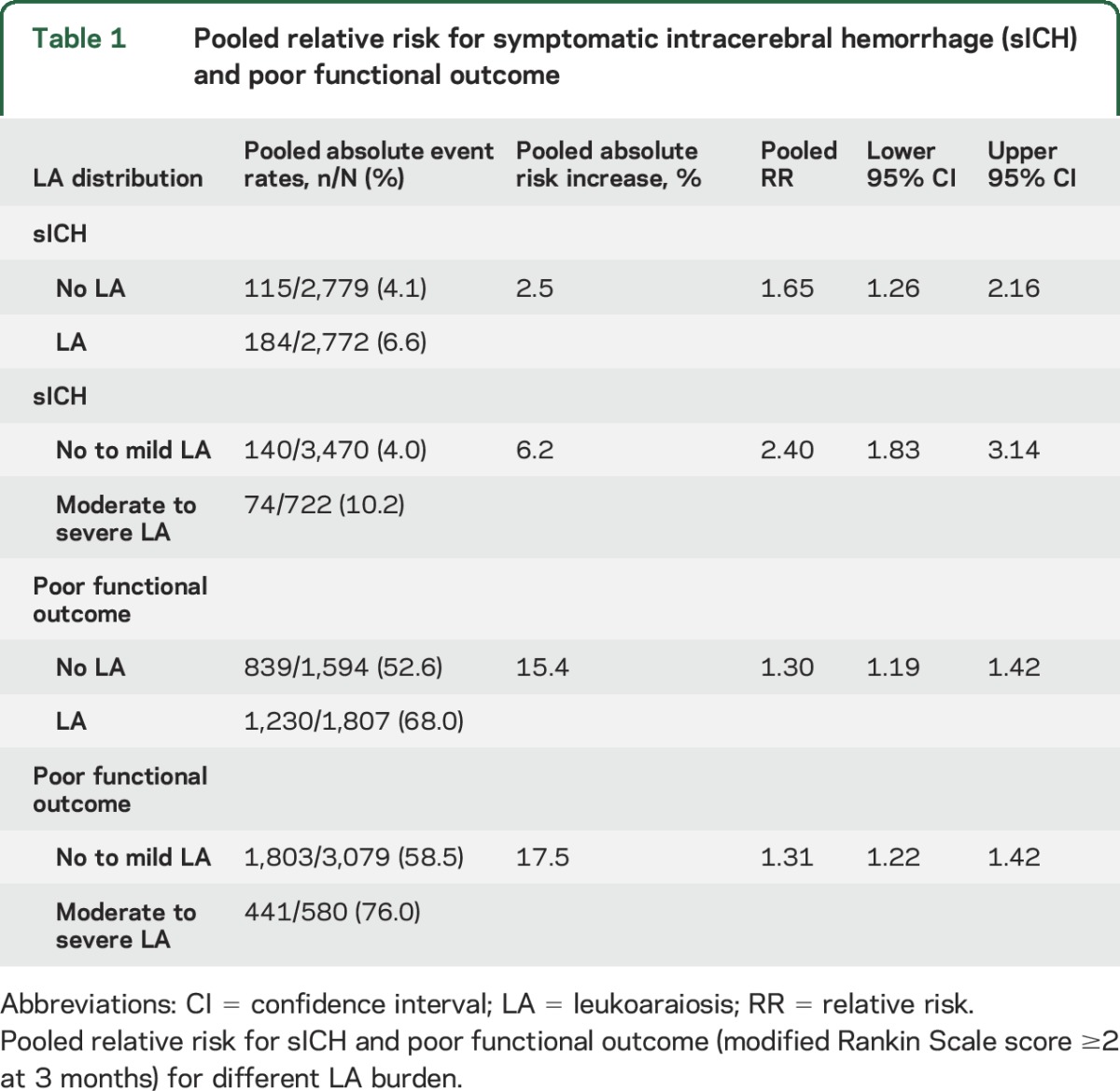
Pooled relative risk for symptomatic intracerebral hemorrhage (sICH) and poor functional outcome

**Figure 2:**
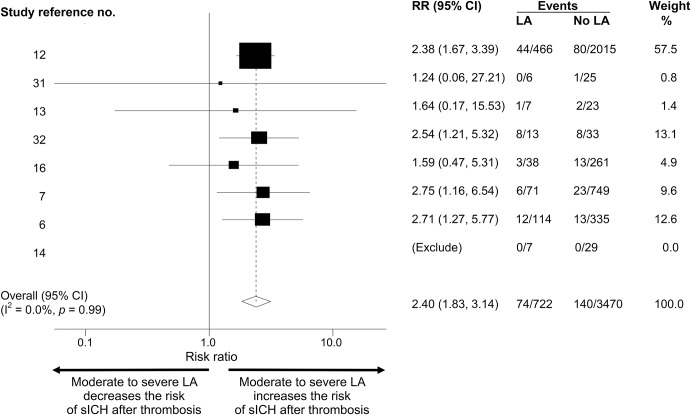
Forest plot of symptomatic intracerebral hemorrhage (sICH): Moderate to severe leukoaraiosis (LA) vs no to mild LA Forest plot of sICH-associated LA in post-thrombolysis patients: moderate to severe LA vs no to mild LA. CI = confidence interval; RR = relative risk.

Eight studies^6,7,9,13,14,16,31,32^ including 4,192 patients compared sICH risk post thrombolysis between patients with no to mild LA and moderate to severe LA. The total sICH rate was 214/4,192 (5.1%). The sICH event rate in those with moderate to severe LA was 10.2% vs 4.0% for those no to mild LA; thus, moderate to severe LA confers an absolute risk increase of 6.2% for sICH (table 1). The pooled RR of sICH risk post thrombolysis was 2.40 (95% CI 1.83–3.14; *p* = 0.001) for those with moderate to severe vs no to mild LA (figure 2). There was no significant heterogeneity between studies (I^2^ was 0%). There was no evidence of publication bias in the funnel plot (figure e-2).

### Functional outcome.

For this analysis, data were available from 11 studies including a total of 5,823 patients. A summary of the characteristics of included studies is provided in tables e-1–e-5. Of the 11 available studies, 9^7,9,13–16,18,31,33^ provided data on poor functional outcome as modified Rankin Scale (mRS) score ≥2 at 3 months. Two articles provided different outcomes (the ISTc group^17^ reported the Oxford Handicap scale ≥3 at 6 months and Shi et al.^32^ reported mRS ≥3 at discharge) so could not be included in the meta-analysis.

**Figure 3:**
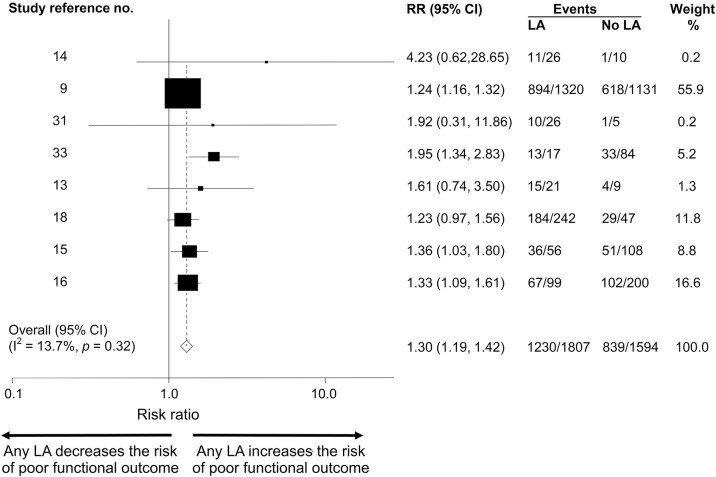
Forest plot of poor functional outcome: Any leukoaraiosis (LA) vs no LA Forest plot of modified Rankin Scale score ≥2 at 3 months associated with LA in post-thrombolysis patients: any LA vs no LA. CI = confidence interval; RR = relative risk.

Eight articles, including 3,401 patients, compared 3-month outcome between patients with no LA vs any LA. The total poor functional outcome rate was 2,069/3,401 (60.8%). The poor functional outcome event rate in those with any LA was 68.0% vs 52.6% for those without LA; thus, any LA confers an absolute risk increase of 15.4% for poor functional outcome (table 1). The pooled RR of poor functional outcome was 1.30 (95% CI 1.19–1.42; *p* = 0.001) for those with any LA vs those without (figure 3). Heterogeneity (I^2^) was 13.7% between the studies. In meta-regression, 3 factors accounted for heterogeneity as assessed by I^2^: hypertension, use of endovascular treatment, and initial stroke severity (NIH Stroke Scale score). However, none of these variables was statistically associated with clinical outcome at 3 months. There was some evidence of publication bias in the funnel plot (figure e-2).

**Figure 4:**
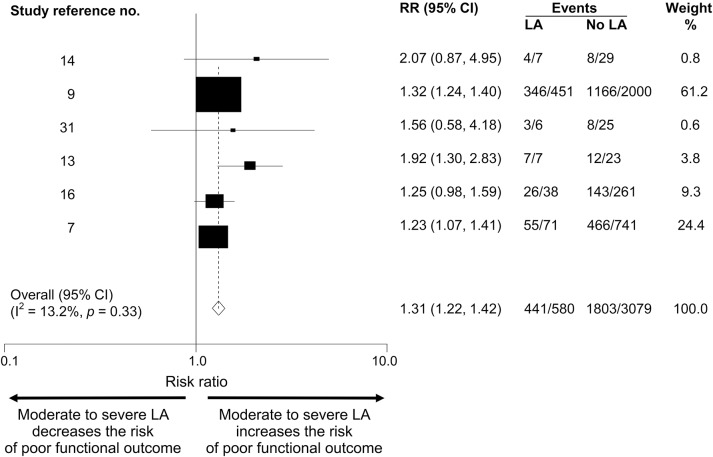
Forest plot of poor functional outcome: Moderate to severe leukoaraiosis (LA) vs no to mild LA Forest plot of modified Rankin Scale score ≥2 at 3 months associated with LA in post-thrombolysis patients: moderate to severe LA vs no to mild LA. CI = confidence interval; RR = relative risk.

Six articles, including 3,659 patients, compared outcome at 3 months between no to mild LA and moderate to severe LA. The total poor functional outcome rate was 2,244/3,659 (61.3%). The poor functional outcome event rate in those with moderate to severe LA was 76.0% vs 58.5% for those no to mild LA; thus, moderate to severe LA confers an absolute risk increase of 17.5% for poor functional outcome (table 1). The pooled RR of poor functional outcome was 1.31 (95% CI 1.22–1.42; *p* = 0.001) for those with moderate to severe LA vs no to mild LA (figure 4). Heterogeneity (I^2^) was 13.2% between the studies. In meta-regression, 2 factors accounted for heterogeneity as assessed by I^2^: hypertension and sex. However, neither of these variables was statistically associated with clinical outcome at 3 months. There was no evidence of publication bias in the funnel plot (figure e-2).

## DISCUSSION

Our systematic review and meta-analysis shows that the presence of LA on pretreatment neuroimaging is associated with an increased risk of sICH and poor functional outcome following thrombolytic treatment. Pooled analyses show that both presence and greater severity of LA are associated with a greater risk of sICH.

Our findings have biological plausibility. LA indicates presumed ischemic white matter injury in the territory of the penetrating small vessels in the distal deep arterial or arteriolar territories, associated with chronic endothelial dysfunction, and disruption of the blood–brain barrier.^34^ The increased risk of sICH after thrombolysis may be explained by damage to the cerebral microcirculation causing increased small vessel fragility and susceptibility to vessel rupture and bleeding complications.^11^ In addition, LA is associated with platelet hyperactivation and hypercoagulability,^35^ which might reduce tissue perfusion after thrombolysis, leading to increased hemorrhagic transformation of the infarct.

Our pooled analyses show that both presence and greater severity of LA are associated with a poor functional outcome. LA might plausibly be relevant for stroke functional recovery: it is associated with cortical neuronal death, white matter disconnection that might result in decreased cerebral plasticity, and impaired learning during neurorehabilitation.^36^ Intact network connectivity of the brain is a critical factor for recovery from ischemic damage after acute stroke,^15^ which might be impaired by LA causing demyelination, loss of axons and oligodendrocytes, and astrocytic gliosis.^37^ Thus, due to these multiple mechanisms, LA could be related to increased risk of haemorrhage, delayed recovery, and impaired plasticity with poorer poststroke functional outcome.^37^ LA is also a risk factor for developing poststroke cognitive impairment, depression, difficulties in walking, and urinary incontinence that may eventually adversely affect patients' compliance with treatment and recovery programs.^38^

Our study has several strengths. We included a large number of patients. We undertook meta-regression to assess for heterogeneity and confounding. Furthermore, undertaking separate meta-analyses on no vs any LA and no to mild vs moderate to severe LA allows us to demonstrated a dose-response effect with a higher risk of sICH in those with more severe LA, which supports a biologically plausible causal association. Finally, our results are consistent with those from studies excluded from the meta-analysis as they measured functional outcome with different scales or time points.

There are some limitations of our analyses. First, the neuroimaging varied between studies, and included both CT and MRI, which is likely to affect the prevalence of LA. Nevertheless, our study shows that medium to high scores on ordinal scales indicating moderate to severe LA (easily detected on CT) carry a high risk of sICH risk compared to those with no to mild LA. This suggests that CT is sufficient to detect clinically relevant LA associated with increased sICH risk and poor functional outcome. Second, there was heterogeneity in thrombolysis protocols and the availability of some baseline characteristics; for example, hypertension, hyperglycemia, prior use of antiplatelet and anticoagulant medications, or recurrent stroke. Third, definitions in sICH varied, although we partially accounted for this heterogeneity by meta-regression. Fourth, there is likely to be selection bias in studies using MRI since unwell patients are more likely to be excluded, although the use of CT or MRI did not appear to be associated with any significant difference in the occurrence of sICH or poor functional outcome. Finally, we have not investigated the effect of ethnicity, which might be relevant for LA prevalence and sICH risk.

The poorer functional outcome in those with LA is likely to be due in part to the increased risk of sICH, particularly in those with moderate to severe LA, in whom the absolute risk was 6.2% higher than in those with no to mild LA. This association begs the question of whether thrombolysis should be withheld in patients with moderate to severe LA. All patients in our analysis were treated with thrombolysis, so we do not know the outcome in patients with or without LA where thrombolysis was withheld based on clinical judgement (which might have included an assessment of severe LA), so we cannot assess for interaction between LA and thrombolysis. In other words, the current study cannot determine whether those with moderate to severe LA would still derive benefit from thrombolysis treatment. Accordingly, our findings do not justify withholding thrombolytic therapy in patients with LA. Nevertheless, subanalysis of a randomized controlled trial shows that the beneficial effect of thrombolysis on favorable functional outcome (mRS 0–1) was only experienced in those without LA.^16^ Thus, our results might justify the need for future randomized control trials (RCTs) of thrombolysis to include stratification of patient outcomes by pretreatment LA. Until such time, other treatment options for those with moderate to severe LA with concern about high sICH risk include endovascular treatment (where a small unadjusted case series saw no difference in sICH rates according to LA grade)^39^ or lower dose tissue plasminogen activator (tPA),^40^ although there is currently no clear evidence supporting the latter strategy; subgroup analyses of the interaction of tPA dose and pretreatment LA in a recent RCT^40^ will be of considerable interest.

Our analysis suggests an approximate doubling of the risk of sICH after thrombolysis for acute ischemic stroke if LA is present on pretreatment neuroimaging; the risk is highest for moderate to severe LA, which is easily detected on plain CT images. Importantly, LA is also associated with increased risk of poor functional outcome after thrombolysis therapy. However, in the absence of randomized trial data, our results do not justify withholding thrombolytic therapy from otherwise eligible candidates solely on the basis of LA presence on neuroimaging. Nevertheless, these data may be useful for clinicians in assessing likely sICH risk and functional outcome in individual patients, and for counseling patients and families about the expected outcome of thrombolytic therapy.

## Supplementary Material

Data Supplement

Accompanying Editorial

## GLOSSARY

CI: confidence interval
ICH: intracerebral hemorrhage
LA: leukoaraiosis
mRS: modified Rankin Scale
RCT: randomized control trial
RR: relative risk
sICH: symptomatic intracerebral hemorrhage
tPA: tissue plasminogen activator
