# Supplementary material for: Leukoaraiosis, intracerebral hemorrhage, and functional outcome after acute stroke thrombolysis
Source: Neurology. 2017 Feb 14;88(7):638–45. doi: 10.1212/WNL.0000000000003605 (PMC5317383; doi:10.1212/WNL.0000000000003605)
Supplement: Data Supplement [file supp_WNL.0000000000003605_supp_file_Figure_e-1.docx]

**Online supplement**

**Figure e-1** Flow chart of study selection

Records identified through database searching

(n = 1,118)

Records after duplicates removed

(n = 667)

Records screened

(n = 667)

Records excluded

(n = 618)

Full-text articles assessed for eligibility

(n = 49)

Studies included in qualitative synthesis

(n = 19)

Studies included in quantitative synthesis (meta-analysis)

(n = 15)

Full-text articles excluded

(n = 30)

Different patient populations (18)

Reviews/Letters (9)

Case reports (1)

Could not construct 2x2 table (2)

**Identification**

**Screening**

**Eligibility**

**Included**

Unavailable outcome after email

(4)
