# Supplementary material for: Leukoaraiosis, intracerebral hemorrhage, and functional outcome after acute stroke thrombolysis
Source: Neurology. 2017 Feb 14;88(7):638–45. doi: 10.1212/WNL.0000000000003605 (PMC5317383; doi:10.1212/WNL.0000000000003605)
Supplement: Data Supplement [file supp_WNL.0000000000003605_supp_file_Figure_e-2.docx]

**Online supplement**

**Figure e-2** Funnel plot

**B**

**D**

**C**

**A**

Symptomatic intracecerebral haemorrhage associated leukoaraiosis (LA) in post-thrombolysis patients:

A) any LA vs none LA B) moderate to severe LA vs none to mild LA

Modified Rankin Score (mRs) > 2 at 3 month associated leukoaraiosis (LA) in post-thrombolysis patients:
C) any LA vs none LA D) moderate to severe LA vs none to mild LA
