# Supplementary material for: Leukoaraiosis, intracerebral hemorrhage, and functional outcome after acute stroke thrombolysis
Source: Neurology. 2017 Feb 14;88(7):638–45. doi: 10.1212/WNL.0000000000003605 (PMC5317383; doi:10.1212/WNL.0000000000003605)
Supplement: Data Supplement [file supp_WNL.0000000000003605_supp_file_Table_e-1_no_HL.docx]

**Table e-1** Characteristics of included studies

| **Reference number** | **Study design** | **Patient number** | **Age**  **(mean)** | **Male sex**  **(%)** | **Initial**  **NIHSS**  **(mean/median)** | **HT (%)** | **AF**  **(%)** | **BS**  **(mmol/l)** | **Anti-platelet (%)** | **Anti- coagulant (%)** | **LA grading scale** | **Neuro imaging** | **Treatment** | **sICH definition** | **Functional outcome** |
| --- | --- | --- | --- | --- | --- | --- | --- | --- | --- | --- | --- | --- | --- | --- | --- |
| 14 | Retrospective single center | 36 | 70 | 78 | 4 | 78 | 33 | 7.3 | 56 | 8 | Van Swieten | CT | IV rt-PA | Any neurologic deterioration < 24 h + hemorrhage  (ECASS) | mRs score > 2 at 3 month |
| 12, 9 | Retrospective single center | 2481  2451* | 69 | 57 56* | 8 | 58 | 26 | 7 | 15 | 6 | Gorter , Van Swieten, Blennow, Wahlund | CT (MRI 3 pt) | IV rt-PA + endovascular  treatment | Any neurologic deterioration or inc NIHSS > 4 at 22-36 h and day 7 + hemorrhage (ECASS II) | mRs score > 2 at 3 month |
| 30 | Retrospective single center | 311 | 68 | - | 7 | - | - | 6 | - | - | Wahlund | CT | IV rt-PA | Inc NIHSS > 4, <36 h + hemorrhage (ECASS I) | - |
| 17 | Retrospective multicenter | 1507 1  1510 2 | - | - | - | - | - | - | 49 | - | Van Swieten,  Fazekas | CT or MRI | IV rt-PA; placebo | Any neurologic deterioration or death within day 7 + hemorrhage | Oxford Handicap scale > 3 at 6 month |
| 31 | Retrospective multicenter | 31 | 76 | 61 | 9 | 84 | 36 | 7.7 | 48 | 3 | Van Swieten | CT | IV rt-PA | Any neurologic deterioration < 24 h + hemorrhage  (ECASS) | mRs score > 2 at 3 month |
| 13 | Retrospective multicenter | 30 | 69 | 77 | 16 | 83 | 23 | 6.6 | 37 | 3 | Van Swieten | CT | IV rt-PA + endovascular  treatment | Any neurologic deterioration < 24 h + hemorrhage  (ECASS) | mRs score > 2 at 3 month |
| 32 | Retrospective single center | 46  103* | 66 | 41 | 18 | 66 | 42 | 7 | 31 | 10 | Fazekas and Schmidt | MRI | Mechanical thrombectomy ± IA rt-PA ± IV rt-PA | Any neurologic deterioration or inc NIHSS > 4 at 24 h and day 6-8 + hemorrhage (ECASS, ECASS II) | mRs score > 3 at discharge |
| 18 | Retrospective single center | 292  289* | 64 | 55 | 15 | 59 | 30 | - | - | - | Fazekas and Schmidt, Scheltens | MRI | IA urokinase ±Mechanical thrombectomy | Inc NIHSS > 4, at 24-72 h + hemorrhage (PROACT II ) | mRs score > 2 at 3 month |
| 15 | Retrospective single center | 164 | 64 | 65 | 13 | 63 | - | 8 | - | - | Fazekas | MRI | IV rt-PA ± IA recombinant pro-urokinase | Inc. NIHSS > 4, <24 h + hemorrhage  (ECASS II) | mRs score > 2 at 3 month |
| 11 | Retrospective single center | 400 | 68 | 53 | 13 | 45 | 23 | 7 | 28 | - | Van Swieten | CT | IV rt-PA | PH2 at 22-36 h and inc ≥4 NIHSS or death < 24 h  (SIS-MOST) | - |
| 16 | Retrospective multicenter | 299^1^  304^2^ | - | - | 14 | - | - | - | - | - | Van Swieten | CT | IV rt-PA; placebo | Any neurologic deterioration < 24 h + hemorrhage  (NINDS) | mRs score > 2 at 3 month |
| 7 | Retrospective multicenter | 820  812* | 70 | 43 | 14 | 53 | 23 | 7 | 21 | - | Van Swieten | CT | IV rt-PA | Any neurologic deterioration < 24 h + hemorrhage  (ECASS) | mRs score > 2 at 3 month |
| 6 | Retrospective multicenter | 449 | 65 | - | 13 | - | - | 7 | - | - | Fazekas and Schmidt | MRI | IV rt-PA ± IA rt-PA or urokinase | Any neurologic deterioration < 36 h + hemorrhage  (NINDS) | - |
| 33 | Retrospective double center | 101 | 66 | 51 | 14 | 68 | 33 | 6 | 6 | - | Not reported | CT | IV rt-PA | - | mRs score > 2 at 3 month |

1 = treatment group; 2 = placebo group; AF = atrial fibrillation; BS = blood sugar; CT = computed tomography; DM = diabetes mellitus; DWI = diffusion-weighted imaging; DWM = deep white matter; HI = haemorrhagic infarction; HT = hypertension; IA = intraarterial; inc = increase; IV = intravenous; LA = leukoaraiosis; MRI = magnetic resonance imaging; mRs = modified Rankin score; NIHSS = national Institutes of Health Stroke Scale; PH = parenchymal haemorrhage; PH2 = parenchymal haemorrhage type2 (SIS-MOST); rt-PA = recombinant tissue plasminogen activator; sICH = symptomatic intracerebral haemorrhage; VSS = van Swieten scale, * = provide different number of patient for functional outcome result
