# Supplementary material for: Leukoaraiosis, intracerebral hemorrhage, and functional outcome after acute stroke thrombolysis
Source: Neurology. 2017 Feb 14;88(7):638–45. doi: 10.1212/WNL.0000000000003605 (PMC5317383; doi:10.1212/WNL.0000000000003605)
Supplement: Data Supplement [file supp_WNL.0000000000003605_supp_file_Table_e-2_no_HL.docx]

**Online supplement**

**Table e-2** Summary of study quality indicators

| **Reference number** | **Study size** | **Clear definition of study population** | **Standardised CT or MRI parameters** | **LA criteria clearly defined** | **sICH criteria clearly defined** | **Standardised rating scale or trained observer agreement reported (inter/intra- rater)** | **Results adjusted for other baseline risk factors** | **No. of quality indicators fulfilled for sICH analysis** | **No. of quality indicators fulfilled for sICH analysis** |
| --- | --- | --- | --- | --- | --- | --- | --- | --- | --- |
| 14 | 36 | YES | YES | YES | YES | YES | YES | 6/6 | 5/5 |
| 12, 9 | 2481  2451* | YES | NO | YES | YES | YES | YES | 5/6 | 4/5 |
| 30 | 311 | YES | YES | YES | YES | NO | NO | 4/6 | - |
| 17 | 1507 1  1510 2 | YES | YES | YES | YES | YES | NO | 5/6 | 4/5 |
| 31 | 31 | YES | YES | YES | YES | YES | YES | 6/6 | 5/5 |
| 13 | 30 | YES | YES | YES | YES | YES | YES | 6/6 | 5/5 |
| 32 | 46  103* | YES | YES | YES | YES | NO | YES | 5/6 | 4/5 |
| 18 | 292  289* | YES | YES | YES | YES | NO | NO | 4/6 | 3/5 |
| 15 | 164 | YES | YES | YES | YES | NO | NO | 4/6 | 3/5 |
| 11 | 400 | YES | YES | YES | YES | NO | YES | 5/6 | - |
| 16 | 299^1^  304^2^ | YES | YES | YES | YES | NO | YES | 5/6 | 4/5 |
| 7 | 820  812* | YES | YES | YES | YES | YES | YES | 6/6 | 5/5 |
| 6 | 449 | YES | YES | YES | YES | YES | YES | 6/6 | - |
| 33 | 101 | YES | NO | YES | - | NO | YES | - | 3/5 |

1 = treatment group; 2 = placebo group; CT = computed tomography; LA = leukoaraiosis; MRI = magnetic resonance imaging; sICH = symptomatic intracerebral haemorrhage, * = provide different number of patient for functional outcome result
