# Supplementary material for: Leukoaraiosis, intracerebral hemorrhage, and functional outcome after acute stroke thrombolysis
Source: Neurology. 2017 Feb 14;88(7):638–45. doi: 10.1212/WNL.0000000000003605 (PMC5317383; doi:10.1212/WNL.0000000000003605)
Supplement: Data Supplement [file supp_WNL.0000000000003605_supp_file_Table_e-3_no_HL.docx]

**Online supplement**

**Table e-3** Details of the thrombolysis treatment protocols of included studies

| **Reference number** | **Study Thrombolysis** |
| --- | --- |
| 14. | IV-tPA dosage: 0.9 mg/kg bodyweight; maximum dose: 90 mg  (NINDS) |
| 12, 9 | Patients were treated based on their departments written guidelines for acute stroke, which are updated biannually and any time when new scientific evidence becomes available. (Guideline: FDA 1996, FDA Warning, AHA Guidelines 2007, EMEA 2002, Helsinki 1998, Helsinki 2005) |
| 30 | IV-tPAwas administered within 4.5 hours from symptom onset according to general guidelines. |
| 17 | IV tPA dosage: 0.9 mg/kg bodyweight; maximum dose: 90 mg |
| 31 | IV-tPA dosage: 0.9 mg/kg bodyweight; maximum dose: 90 mg  (NINDS) |
| 13 | IV-tPA dosage: 0.9 mg/kg bodyweight; maximum dose: 90 mg  (NINDS) |
| 32 | Patients treated by thrombectomy were either ineligible for IV-tPA or the occluded vessel failed to recanalize after receiving IV-tPA (0.9 mg/kg) within 3 hours of stroke onset. IA-tPA was allowed as an adjunct to thrombectomy. Rescue intracranial angioplasty or stenting was also performed after failed thrombectomy. Carotid stenting was allowed in cases with proximal stenosis or dissection after thrombectomy. |
| 18 | The interventional neuroradiologists decided on the use of urokinase, mechanical intervention, or both as recanalization techniques. |
| 15 | IV-tPA dosage: 0.9 mg/kg bodyweight; maximum dose: 90 mg  (NINDS) |
| 11 | IV-tPA dosage: 0.9 mg/kg bodyweight; maximum dose: 90 mg  (NINDS, ECASS II) |
| 16 | IV tPA dosage: 0.9 mg/kg bodyweight; maximum dose: 90 mg |
| 7 | IV tPA dosage: 0.9 mg/kg bodyweight; maximum dose: 90 mg |
| 6 | (1) IV tPA treatment (n=363; treatment within 3 hours, 69%) and (2) IA or combined IV/IA treatment with either tPA or urokinase (n=86; treatment within 3 hours, 30%) |
| 33 | IV tPA dosage: 0.9 mg/kg bodyweight; maximum dose: 90 mg |

ECASS II = European Cooperative Acute Stroke Study–II; IA-tPA = Intra-arterial tissue plasminogen activator; IV-tPA = intravenous tissue plasminogen activator; NINDS = National Institute of Neurological Disorders Study.
