# Supplementary material for: Leukoaraiosis, intracerebral hemorrhage, and functional outcome after acute stroke thrombolysis
Source: Neurology. 2017 Feb 14;88(7):638–45. doi: 10.1212/WNL.0000000000003605 (PMC5317383; doi:10.1212/WNL.0000000000003605)
Supplement: Data Supplement [file supp_WNL.0000000000003605_supp_file_Table_e-4_no_HL.docx]

**Online supplement**

**Table e-4** Post rtPA haemorrhage as defined in important clinical trials

| 1. ECASS II   Symptomatic intracranial haemorrhage was defined as blood at any site in the brain on the Computed tomography (CT) scan (as assessed by the CT reading panel, independently of the assessment by the investigator), documentation by the investigator of clinical deterioration, or adverse events indicating clinical worsening (eg, drowsiness, increase of hemiparesis) or causing a decrease in the National Institutes of Health Stroke Scale (NIHSS) score of 4 or more points. Scans at 22-36 hr and day 7.  Hacke W, Kaste M, Fieschi C, von Kummer R, Davalos A, Meier D, et al. Randomised double-blind placebo-controlled trial of thrombolytic therapy with intravenous alteplase in acute ischaemic stroke (ECASS II). Second European-Australasian Acute Stroke Study Investigators. *Lancet* 1998;352:1245–1251.   1. ECASS I   Hemorrhagic events were classified as Haemorrhagic infarction (HI) types I and II and Parenchtmal haemorrhage (PH) types I and II. HI I is defined as small petechiae along the margins of the infarct, while HI II represents more confluent petechiae within the infracted area, but without space-occupying effect. PH I is defined as blood clot not exceeding 30% of the infarcted area with some mild space-occupying effect, and PH II represents dense blood clot(s) exceeding 30% of the infarct volume with significant space-occupying effect. CT at 24 hrs and 6-8 days and discretion of investigator.  Fiorelli M, Bastianello S, Von Kummer R, et al. Hemorrhagic transformation within 36 hours of a cerebral infarct relationships with early clinical deterioration and 3-month outcome in the European Cooperative Acute Stroke Study I (ECASS I) Cohort. Stroke 1999;30:2280-2284.   1. ECASS   Hemorrhagic events were classified as HI types I and II and PH types I and II. HI I is defined as small petechiae along the margins of the infarct, while HI II represents more confluent petechiae within the infracted area, but without space-occupying effect. PH I is defined as blood clot not exceeding 30% of the infarcted area with some mild space-occupying effect, and PH II represents dense blood clot(s) exceeding 30% of the infarct volume with significant space-occupying effect. CT at 24 hrs and 6-8 days and discretion of investigator.  Hacke W, Kaste M, Fieschi C, Toni D, Lesaffre E, von Kummer R, Boysen G, Bluhmki E, Hoxter G, Mahagne M. Intravenous thrombolysis with recombinant tissue plasminogen activator for acute hemispheric stroke: the European Cooperative Acute Stroke Study (ECASS).JAMA 1995; 274: 1017-1025.   1. PROACT II   The presence of intracerebral haemorrhage (ICH) with neurologic deterioration, defined as an increase of four or more points in the NIHSS score in comparison with the pre-angiography score, within 36 hours from treatment initiation.  Kase CS, Furlan AJ, Wechsler LR, Higashida RT, Rowley HA, Hart RG, Molinari GF, Frederick LS, Roberts HC, Gebel JM, Sila CA, Schulz GA, Roberts RS, Gent M (2001) Cerebral hemor- rhage after intra-arterial thrombolysis for ischemic stroke: the PROACT II trial. Neurology 57:1603–1610.   1. SITS-MOST   Symptomatic ICH, per the SITS-MOST protocol, was defined as local or remote PH type 2 on the 22–36 h post-treatment imaging scan, combined with a neurological deterioration of 4 points or more on the NIHSS from baseline, or from the lowest NIHSS value between baseline and 24 h, or leading to death.  Wahlgren N, Ahmed N, Davalos A, et al. Thrombolysis with alteplase for acute ischaemic stroke in the Safe Implementation of Thrombolysis in Stroke-Monitoring Study (SITS-MOST): an observational study. Lancet 2007; 369: 275–282.   1. NIND   Intracranial hemorrhage, serious systemic bleeding, death, and new stroke were the primary adverse events monitored. To detect intracranial hemorrhage, CT scans were required at 24 hours and 7 to 10 days after the onset of stroke and when any clinical finding suggested hemorrhage. A hemorrhage was considered symptomatic if it was not seen on a previous CT scan and there had subsequently been either a suspicion of hemorrhage or any decline in neurologic status.  The National Institute of Neurological Dis- orders and Stroke rt-PA Stroke Study Group: Tissue plasminogen activator for acute ischemic stroke. N Engl J Med 1995;333:1581– 1587. |
| --- |
