# Supplementary material for: Leukoaraiosis, intracerebral hemorrhage, and functional outcome after acute stroke thrombolysis
Source: Neurology. 2017 Feb 14;88(7):638–45. doi: 10.1212/WNL.0000000000003605 (PMC5317383; doi:10.1212/WNL.0000000000003605)
Supplement: Data Supplement [file supp_WNL.0000000000003605_supp_file_Table_e-5_no_HL.docx]

**Online supplement**

**Table e-5** Ideal design of brain imaging study to investigate the presence of leukoaraiosis

| Study population:  􏰢  Clearly defined clinical characteristics and selection criteria  􏰢  Representative of the population of patients with acute ischaemic stroke (consecutive cases in centres using CT or MRI routinely for acute stroke assessment)  􏰢  Report of number and characteristics of patients excluded |
| --- |
| Detection and rating of LA:   Standardized CT or MRI parameters for T2 weighted imaging (field strength, echo time, slice thickness, gap, etc.)  􏰢  Clear definition of LA  􏰢  Use of a standardized LA rating instrument with clearly defined anatomical regions   Rating instrument demonstrated to have good inter- and intra-rater reliability trained observers (ideally a single observer for all analysis in a study). |
| Definition of outcome:  􏰢  Post-thrombolysis ICH definition criteria and methods and timing of assessment clearly defined  􏰢  Clinically relevant definition of post-thrombolysis ICH (e.g. associated with significant clinical deterioration)  􏰢 Reporting and analysis: Results adjusted for confounding from other baseline risk factors known to be associated with thrombolysis-related ICH and LA (including age, early ischaemic CT changes, high blood pressure, hyperglycaemia, increasing stroke severity, etc.)  􏰢  Results presented according to severity of LA |

CT = computed tomography; LA = leukoaraiosis; MRI = magnetic resonance imaging; ICH = intracerebral haemorrhage
