# Supplementary material for: Leukoaraiosis, intracerebral hemorrhage, and functional outcome after acute stroke thrombolysis
Source: Neurology. 2017 Feb 14;88(7):638–45. doi: 10.1212/WNL.0000000000003605 (PMC5317383; doi:10.1212/WNL.0000000000003605)
Supplement: Accompanying Editorial [file supp_88_7_638_v2_index.html]

Accompanying Editorial 

# Leukoaraiosis, intracerebral hemorrhage, and functional outcome after acute stroke thrombolysis

## Accompanying Editorial

**Neurology® data supplements are not copyedited before publication. Published editorials and translations have been copyedited.  
 © 2017 American Academy of Neurology.  
  
 Files in this Data Supplement:**

- Accompanying Editorial - PDF
