# Supplementary material for: Leukoaraiosis, intracerebral hemorrhage, and functional outcome after acute stroke thrombolysis
Source: Neurology. 2017 Feb 14;88(7):638–45. doi: 10.1212/WNL.0000000000003605 (PMC5317383; doi:10.1212/WNL.0000000000003605)
Supplement: Accompanying Editorial [file supp_WNL.0000000000003605_612.pdf]

EDITORIAL

Thrombolysis for acute ischemic stroke in patients with leukoaraiosis

Caution needed

Jonathan Streifler, MD
Pauline Maillard, PhD

Correspondence to Dr. Streifler: streifll@post.tau.ac.il

Neurology® 2017;88:612–613

The term leukoaraiosis (LA), introduced about 30 years ago,[1] describes cerebral white matter signal abnormalities (or white matter hyperintensities) commonly observed on structural brain magnetic resonance or CT imaging in elderly individuals. LA severity can be graded using categorical classification, based on different rating scales,[2] or continuously, using automatic quantification algorithms. LA has multiple histopathologic correlates, including ependymal loss, cerebral ischemia, demyelination, venous collagenosis, and microcystic infarcts.[3] A substantial body of evidence now supports associations between LA and increased risk for motor disability, dementia, and mortality,[4] as well as an increased risk for future stroke or TIA.[5] LA strongly associates with increasing age and vascular risk factors.[6] Particularly, recent studies found associations between LA extent and increased risk for symptomatic intracerebral hemorrhage (sICH) with thrombolytic agents,[7] as well as with the use of antithrombotic agents and anticoagulants.[8] Yet in spite of overwhelming evidence of such associations, LA is not considered as a relevant indicator in the assessment checklists for consideration of the use of thrombolytic agents after an acute ischemic stroke (AIS).[9]

In a comprehensive systematic review and meta-analysis published in this issue of *Neurology*®, Kongbunkiat et al.[10] found that, among patients treated with thrombolysis after an AIS, those with greater severity of LA had higher risk for sICH post thrombolysis as well as worse functional outcome as modified Rankin Scale score >2 at 3 months. Interestingly, such findings depended on how LA severity groups (none, mild, moderate and severe) were combined. Thus, when LA severity groups included presence of LA vs no LA, relative risk (RR) was 1.3 and 1.65 for functional outcome and sICH, respectively. When dichotomized as moderate and severe LA vs no and mild LA, the RR remained unchanged for functional outcome (1.31) but reached 2.4 for sICH (equivalent to an absolute risk increase of 6.2%, with 10.2% vs 4.0%). This finding suggests that the increase in the risk of brain hemorrhage in presence of even moderate LA (as compared to no and mild LA) may overcome the benefit of thrombolysis. This observation emphasizes the need to determine if screening patients for LA presence and extent before a decision is taken to administer tissue plasminogen activator (tPA) can improve the safety and effectiveness of IV tPA use in AIS.

The study has some notable limitations. First, the lack of a control group for untreated patients makes definitive comparisons difficult. Second, the authors estimated relative risks from different patient samples. The comparison of sICH risk between no vs any LA derived from 10 studies, including 5,551 patients; comparison between no and mild vs moderate and severe LA used only 8 studies, including 4,192 patients. Similarly, functional outcome analyses between no vs any LA were based on 6 studies, including 3,659 patients, while comparison between no and mild vs moderate and severe LA used data from 8 studies, including 3,401 patients. Finally, the lack of standardization of LA severity scales across studies or the specific imaging sequences used to grade LA severity further limits the interpretation of the study.

What next? First, longitudinal studies should confirm these findings and consider how to standardize LA severity estimation using either a gold standard rating scale or using continuous measures of LA burden. Second, efforts to reduce morbidity and lower health care costs through prevention remain crucial and need prioritization. Third, research should focus on the pathologic mechanisms that make individuals with an advanced white matter injury, as reflected by LA, more prone to sICH after being treated by thrombolytic therapy for AIS, and determine if these mechanisms differ from known predictors of poor outcome, such as advanced age, initial stroke severity, and poor blood pressure control.

This meta-analysis conducted by Kongbunkiat et al.[10] has important clinical implications. The investigators convincingly identify a subgroup of patients, i.e., individuals with either moderate or severe LA, for whom thrombolytic agents might be hazardous. Nonetheless, before any recommendation to withhold treatment in this subgroup can be made, randomized control trials

See page 638

From the Department of Neurology (J.S.), Rabin Medical Center, Petah Tikva; the Sackler Faculty of Medicine (J.S.), Tel Aviv University, Israel; and Department of Neurology and Center for Neuroscience (P.M.), Imaging of Dementia and Aging Laboratory, University of California, Davis.
Go to Neurology.org for full disclosures. Funding information and disclosures deemed relevant by the authors, if any, are provided at the end of the editorial.

© 2017 American Academy of Neurology
© 2017 American Academy of Neurology. Unauthorized reproduction of this article is prohibited.

specifically designed to confirm this finding are needed. Meanwhile, clinicians will need guidance for assessment and diagnosis, and alternative options such as lower tPA dose or endovascular approach should be evaluated for this subgroup of patients.

STUDY FUNDING

No targeted funding reported.

DISCLOSURE

The authors report no disclosures relevant to the manuscript. Go to Neurology.org for full disclosures.

REFERENCES

1. Hachinski VC, Potter P, Merskey H. Leukoaraiosis. Arch Neurol 1987;44:21–23.
2. Pantoni L, Simoni M, Pracucci G, Schmidt R, Barkhof F, Inzitari D. Visual rating scales for age-related white matter changes (leukoaraiosis): can the heterogeneity be reduced? Stroke 2002;33:2827–2833.
3. Pantoni L, Garcia JH. Pathogenesis of leukoaraiosis: a review. Stroke 1997;28:652–659.
4. Debette S, Markus HS. The clinical importance of white matter hyperintensities on brain magnetic resonance imaging: systematic review and meta-analysis. BMJ 2010;341:c3666.
5. Streifler JY, Eliasziw M, Benavente OR, et al. Prognostic importance of leukoaraiosis in patients with symptomatic internal carotid artery stenosis. Stroke 2002;33: 1651–1655.
6. Maillard P, Carmichael OT, Reed B, Mungas D, DeCarli C. Cooccurrence of vascular risk factors and late-life white-matter integrity changes. Neurobiol Aging 2015;36: 1670–1677.
7. Palumbo V, Boulanger JM, Hill MD, Inzitari D, Buchan AM; CASES Investigators. Leukoaraiosis and intracerebral hemorrhage after thrombolysis in acute stroke. Neurology 2007;68:1020–1024.
8. Gorter JW. Major bleeding during anticoagulation after cerebral ischemia: patterns and risk factors: Stroke Prevention in Reversible Ischemia Trial (SPIRIT): European Atrial Fibrillation Trial (EAFT) study groups. Neurology 1999;53:1319–1327.
9. Demaerschalk BM, Kleindorfer DO, Adeoye OM, et al. Scientific rationale for the inclusion and exclusion criteria for intravenous alteplase in acute ischemic stroke: a statement for healthcare professionals from the American Heart Association/American Stroke Association. Stroke 2016;47: 581–641.
10. Kongbunkiat K, Wilson D, Kasemsap N, et al. Leukoaraiosis, intracerebral hemorrhage, and functional outcome after acute stroke thrombolysis. Neurology 2017;88:638–645.

© 2017 American Academy of Neurology. Unauthorized reproduction of this article is prohibited.
